# Perceptual and sensorimotor adaptations to hypogravity: implications for manual task performance and verticality perception

**DOI:** 10.3389/fpsyg.2026.1843289

**Published:** 2026-06-30

**Authors:** Tatiana Maillard, Jean-Pierre Bresciani

**Affiliations:** Control and Perception Laboratory, Department of Neuro- and Movement Sciences, University of Fribourg, Fribourg, Switzerland

**Keywords:** perception, upper limb motor control, human space flight program, human-machine interface, postural control, reduced gravity, rehabilitation, sensorimotor adaptation

## Abstract

Hypogravity environments (e.g., 1/6 g on the Moon and 3/8 g on Mars, where gravity is lower than on Earth) profoundly alter the sensorimotor mechanisms underlying spatial orientation, perception, and manual task execution. Understanding these adaptations is essential for ensuring astronaut operational performance. In particular, there is a need for a better understanding of the long-term effects of hypogravity on manual task performance during seated operations, such as piloting, landing, and navigating, which rely on the integration of vestibular, visual, and somatosensory signals that drive motor adaptation in unfamiliar gravitational environments. However, sensorimotor processes and adaptation to hypogravity remain incompletely understood, particularly after prolonged exposure. This perspective paper synthesizes current knowledge largely derived from experimental platforms with inherent constraints. Additionally, it explores the convergence of technological approaches used both to simulate hypogravity for spaceflight preparation and to support rehabilitation after vestibular or neurological impairment. Finally, it suggests that future research should focus on long-term hypogravity simulation using AI-driven assistive technologies through interdisciplinary collaboration.

## 1 Introduction

Hypogravity (HG) is an unfamiliar environment for human physiology. This gravity presents a challenge to human biomechanics due to decreased weight and reduced normal forces, while inertia remains unchanged, and friction is reduced. In parallel, it induces physiological adaptations in sensorimotor, musculoskeletal, and cardiovascular systems, which impact spatial orientation, perception, and task performance (Hides et al., 2017).

Impairment of sensory inputs under altered gravity, such as the proprioceptive and otolithic vestibular, disrupts movement coordination (Dichgans et al., 1976; Nashner, 1976; Glukhikh et al., 2022; Saveko et al., 2020; Schoenmaekers et al., 2022), only partially restored after spaceflight (Saveko et al., 2020), and reduces motor reactions needed for postural control (Kozlovskaya et al., 1988; Tomilovskaya et al., 2019; Shishkin et al., 2023), while sensorimotor disruption is modulated by experience (Schoenmaekers et al., 2022). Accordingly, sensorimotor function has been shown to be significantly affected across a range of gravitational conditions, including during parabolic flight (PF) short-duration weightlessness (Weber et al., 2025; White et al., 2020), during and after short and long duration microgravity exposure (Reschke and Clément, 2018; Wood et al., 2011), ground-based microgravity analogs, such as dry immersion (DI) (Tomilovskaya et al., 2019; Treffel et al., 2016) and head-down bed rest (HDBR) (Mulavara et al., 2018), hypergravity (>1 g) (White et al., 2020; Stahn et al., 2020; Kunavar et al., 2021; Ritzmann et al., 2019), hypogravity 0 < g < 1g (Ritzmann et al., 2019; Maillard, 2023a; Clément et al., 2024).

The multisensory integration of vestibular, visual, and proprioceptive cues allows individuals to construct an internal model of gravity. However, its calibration becomes difficult when sensory information is conflicting (Di Cesare et al., 2014; Zago and Lacquaniti, 2005). HG exposed individuals to an unfamiliar combination of sensory inputs: impairing verticality perception (Treffel et al., 2016; Mulavara et al., 2018; Galvan-Garza et al., 2018; Keime et al., 2023), balance control (Kobel et al., 2021), and movement control (Galvan-Garza et al., 2018; Keime et al., 2023; Maillard, 2023a). These changes can significantly affect fine-motor control tasks that are important for piloting, rover driving, landing, and terrain navigation, including rapid manual control access and smooth trajectory guidance (Paloski et al., 2008). For such critical manual tasks performed in a seated position, effective performance under HG requires processing spatial orientation and stabilizing body posture to achieve precise trunk control (Mergner and Rosemeier, 1998; Swanenburg et al., 2020; Clément et al., 2024). Short-term adaptation occurs more readily in balancing and reaching tasks than during seated tasks requiring accurate verticality perception or spatial updating (Crevecoeur et al., 2009; Gaveau et al., 2011). And transition between gravitational environments requires particular attention due to the associated high operational risks (Shelhamer, 1985).

The changes in sensorimotor systems under HG have been primarily investigated during posture transitions from sitting to standing, manual pointing/tracking, and whole-body balance and locomotion (Weber et al., 2025; White et al., 2020; Stahn et al., 2020; Kunavar et al., 2021; Ritzmann et al., 2019; Clément et al., 2024). In contrast, operational manual tasks in seating position remain underexplored (Maillard, 2023a).

Despite recent advancements, most experimental paradigms have limitations in the duration of HG exposure, making it difficult to study long-term adaptation, particularly in terms of physiological and behavioral adaptations. Therefore, there is a growing need to investigate the impact of HG on humans through long-duration, posture-specific, and task-relevant assessments that mimic operational conditions.

This paper reviews the current understanding of perceptual and motor processes affected in HG and examines how space and rehabilitation science converge to generate new insights. It also offers a perspective on future methodological and technological advancements for both astronaut training and patients with sensorimotor impairments.

## 2 Major findings for perceptual processes under hypogravity

HG primarily affects the following perceptual processes^1^: spatial orientation, visual vertical perception, proprioception and vestibular function, and body schema.

Under HG, the sensorimotor system reweights different sensory inputs so that spatial orientation shifts. The reliance on visual inputs increases as otolith-mediated graviceptive signals decrease (Hilbig et al., 2017; Saveko et al., 2023; Kuldavletova, 2020; Alberts, 2016), making visual inputs the primary signal for resolving conflicting sensory cues (Hilbig et al., 2017; Diaz-Artiles et al., 2022; Harris et al., 2017; Meskers et al., 2021; Clark and Young, 2017; Galvan-Garza et al., 2018).

The reduction in vestibular input from HG drives this compensatory redistribution across the sensorimotor networks (White et al., 2020). During gravity transitions, small left–right or modality asymmetries in the vestibular–graviceptive system imbalances can occur until the sensorimotor system adapts (Alberts, 2016).

The perception of body rotation around the sagittal and lateral axes is an important component of spatial orientation. Under HG, *roll-tilt perception* tends to be underestimated, so that the same tilt feels smaller than under 1 g, a phenomenon called “G-shortage,” causing the visual target to be perceived as higher (Clark and Young, 2017; Godwin, 2000; Galvan-Garza et al., 2018). The Observer model, derived from a computational framework calibrated on human vestibular and sensorimotor response data, predicts a systematic underestimation of tilt, indicating that (~17.5% underestimation for a 20° static roll tilt in 0.5 g) (Godwin, 2000), and even more under at 3/8 g and 1/6 g levels (Galvan-Garza et al., 2018). Although practice improves the accuracy of roll-tilt perception, variability remains high for a prolonged time (Galvan-Garza et al., 2018; Kuldavletova, 2020; Alberts, 2016; Clark and Young, 2017; Clément et al., 2001; Love et al., 1985).

*Subjective visual vertical* (SVV) refers to the perceived upright relative to gravity. Deviations of SVV can reveal misperception of the body’s tilt and orientation. Under HG, SVV shifts toward the trunk due to the degraded reliability of vestibular cues (White et al., 2020; de Winkel et al., 2012; Kuldavletova, 2020) and limited engagement of the insular/vestibular system (White et al., 2020; Kuldavletova, 2020). Interestingly, a critical threshold of about 0.3 g was identified (*n* = 6) during the 20–30 s PF through the (SVV), below which gravitational cues depend on body-centric references rather than the true vertical (de Winkel et al., 2012). Neurovestibular studies using the Neurolab mission centrifugation, tested perceived roll or pitch tilt during pre-flight and during the flight testing (*n* = 4), show that from roll or pitch tilt illusions occur at intermediate g-levels of 0.5–1 g, with fast adaptation over 16 days in MG, but only partial recalibration (Clément et al., 2001). These findings are further supported by experiments showing that long-duration spaceflight induces adaptive changes in otolith-mediated ocular reflexes (*n* = 27) during pre/post-flight centrifugation (Schoenmaekers et al., 2022) and in multisensory integration with fewer alterations in trained astronauts (*n* = 25) using two video oculography systems on the International Space Station (Glukhikh et al., 2022). Below that 0.22 g, verticality perception and task performance become affected. Moreover, variability in distance, slope, and relative position increases, near distances are underestimated, and far distances are overestimated, creating potential risks for balance-related incidents during extravehicular activity (Goswami et al., 2021; Goswami et al., 2012). Moreover, misinterpretation of lunar terrain is also altered by depth perception, altered interpretation of color, contrast, and shading in the absence of an atmosphere, and unfamiliar scale (Goswami et al., 2012; Clément et al., 2008; Bloomberg et al., 2015; Daum and Hecht, 2009).

*Proprioceptive* signals are essential for task performance. Under HG, multiple sources of proprioceptive input are affected. Muscle spindle sensitivity rapidly decreases, and concurrently, the vestibulo-spinal reflex that normally stabilizes posture is also reduced (Hilbig et al., 2017). With less g load on the muscles, joints, and feet, proprioceptive drive can further decrease (Richter et al., 2017) and may initially remain stable across g-transitions due to short-term memory (Weber et al., 2025). Additionally, plantar cutaneous function is also altered: astronauts exhibit frequency-dependent changes in vibration perception after short-duration spaceflight (Shishkin et al., 2023; Kozlovskaya, 2018). This shows an alternation in proprioceptive neural signals, crucial for maintaining postural muscle activity (Kozlovskaya et al., 2007). The same authors state that withdrawal conditions decrease the transverse stiffness and force of postural muscles. However, mechanical stimulation of the plantar zones can prevent these changes. The muscle tone changes have already been reported (*n* = 12) after 2 h of exposure to DI supportlessness (Amirova et al., 2021).

Consequently, proprioceptive input reduction under HG prompts recalibration of the *body schema*, but it remains shaped by prior expectations: as a result, verticality drifts and vestibular reliability declines (White et al., 2020; Clark and Young, 2017). The sensorimotor system incrementally adapts, recalibrating body schema to preserve spatial perception and action (Morfoisse et al., 2024). Adaptation appears to first rely on the internal evaluation of SVV and proprioceptive input, and then gradually rebalance across other sensory cues (Clément et al., 2001; Rock et al., 2024). Adaptation of predictive control may plateau after 6 months of exposure (*n* = 15) to microgravity (MG), followed by recovery to baseline within ~30 days post-flight. (Tays et al., 2021).

## 3 Major findings for motor processes under hypogravity

This section reviews how HG alters motor processes, including sensorimotor planning and control, postural control and balance, reflexes and motor coordination, fine motor control, and spatial updating.

**Figure 1:**
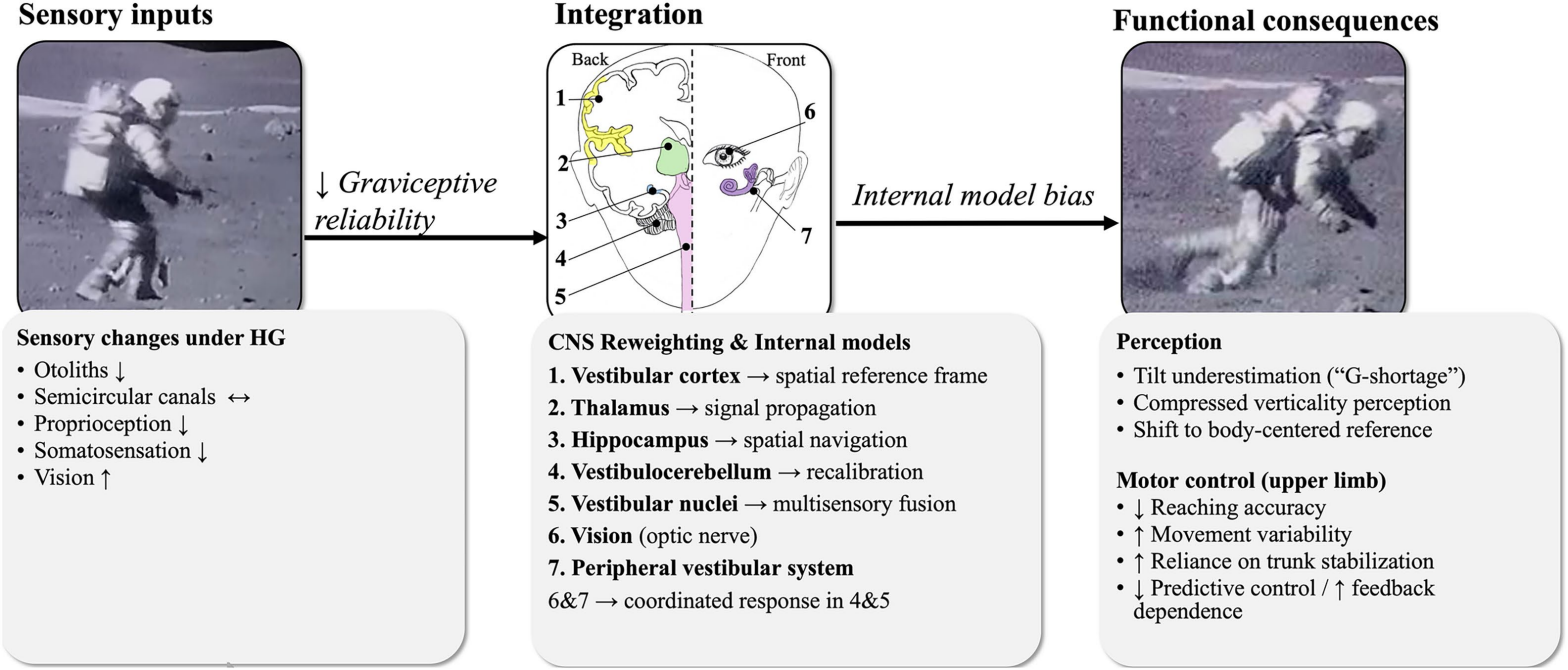
Conceptual model of sensorimotor reweighting mechanism under hypogravity (HG). HG alters sensory reliability (left) → leads to central nervous system (CNS) reweighting (middle) → impairs sensory perception and motor control (right). ↑ ↓ increased/decreased reliance, ↔ unchanged. Apollo astronaut on the Moon. Image credit: NASA.

Research on *sensorimotor planning and control* focuses on the ability to reach for targets or objects located within our peripersonal space. These interactions require encoding the object’s position relative to the body position and orientation, based on visual, proprioceptive, and vestibular signals (Mergner, 2002). In line with this, many studies have demonstrated the role played by vestibular signals when reaching for targets located in the peripersonal space (Blouin et al., 2015), be it after detected changes of body position/orientation (Bresciani et al., 2002a; Bresciani et al., 2005; Guillaud et al., 2006; Blouin et al., 2010; Schomaker et al., 2011; Reichenbach et al., 2016) or after galvanic stimulation of the vestibular system (Bresciani et al., 2002b; Bresciani et al., 2002c; Mars et al., 2003). Thus, under HG, the accuracy of reaching movements decreases (Bock et al., 1992; Watt, 1997), probably because of altered vestibular and proprioceptive information (Bock, 1992; Lackner and DiZio, 1992; Bringoux et al., 2012). See Figure 1, summarizing this process. Moreover, the lack of involvement in proprioceptive muscle spindles under HG (Dietz et al., 1992; Layne et al., 1985; Ritzmann et al., 2015) further reduces the accuracy of sensorimotor control. Thus, control pathways that carry signals from the spinal cord to the brain and back down again to refine movements and reflexes become longer (Mauritz and Dietz, 1980).

HG also significantly impacts *postural control and balance* by shifting posture toward greater reliance on proximal and trunk segments, stiffening the torso, and enabling the trunk to lead balance adjustments (White et al., 2020; Holubarsch et al., 2019; De Martino et al., 1985). This aligns with studies conducted after short- *n* = 10 (Speers et al., 1998) and long-duration *n* = 33 (Shishkin et al., 2023) space flights, which demonstrated that the postural system switches from a more challenging strategy—the ankle—to a less challenging one—the hip. As a result, spinal stiffness increases to stabilize posture (Swanenburg et al., 2023), and arm torque contributes to appropriate motor planning (Bringoux et al., 2012). Also, the Center of Mass balance requires larger-magnitude, slower-paced corrections in hip/trunk control, ensuring stability through a stiffness strategy and greater postural sway (Maillard, 2023b; Ritzmann et al., 2015; Ritzmann et al., 2019), and distal ankle strategies (Ritzmann et al., 2015). PF simulation of HG also demonstrates progressive co-contraction of the lower limb. However, this is an inefficient strategy for fine-tuning capacity (Ritzmann et al., 2015; Nagai et al., 2013). Evidence of postural control and balance issues has been documented in Apollo astronauts, who recorded at least 23 falls during all Moon missions (Goswami et al., 2021; Goswami et al., 2012).

HG also alters the *reflex mechanisms*. Time-course reflexes are shifted, often with lower thresholds and increased T/H-reflex amplitudes (Kozlovskaya et al., 2006; Zakirova et al., 2015). Head-out water immersion and unilateral limb unloading experiments show that reflexes under HG become more excitable or flatten the normal posture-dependent reflex tuning (Kozlovskaya, 2018; Zakirova et al., 2015; Egawa et al., 2000; Clark et al., 1985; Seynnes et al., 2008; Seynnes et al., 2010), making force less smooth and precise, reactions slower or more variable, and muscles more fatigued and weaker.

*Fine motor control* changes were observed under HG. Over time, it decreases muscle strength due to deconditioning caused by changes in neuromuscular coordination and reduced proprioceptive drive (Holubarsch et al., 2019; Sangals et al., 1999). The study of subjective workload during fine motor tasks under HG showed slightly higher values than under 1 g, especially for keyboard tasks and assembly (Volkova et al., 2022). Consistently, precision gradation deteriorates, as fewer force steps and higher force thresholds occur, and joint stiffening limits micro-adjustments under HG (Kozlovskaya, 2018; Hilbig et al., 2017; Holubarsch et al., 2019).

Furthermore, *spatial updating* is also affected and only partially recovers (Love et al., 1985; De Martino et al., 1985; Rosenberg et al., 2018; Volkova, 2022). Early deficits can be notable: roll-tilt manual control performance drops by up to 69% at 0.5 g (*n* = 10; 69% refers to immediate reaction; joystick-based closed-loop manual control task on centrifuge; exposure = 18 min), with only slight improvement after adaptation (Love et al., 1985). Long-duration exposure to altered gravity in HDBR may further inhibit movement and slow reaction times, especially among participants with greater visual reliance (Banker et al., 2021). Moreover, compensatory motor strategies add to the energetic cost of locomotion in HG (Chappell and Klaus, 2013).

## 4 Convergent technologies: space medicine and rehabilitation

Research in human spaceflight and neurorehabilitation converges around a shared problem: sensorimotor alterations and adaptive mechanisms, which provide an opportunity for bidirectional translation. Both fields have developed technologies that modulate body loading and sensory input and share common adaptive processes, including multisensory reweighting, internal model recalibration, and changes in coordination, which may inform future solutions. Current analog platforms differentially modulate gravitational loading, sensory input, and duration, but none provide an integrated framework for long-term investigation of upper-limb sensorimotor adaptation under controlled gravity conditions, see Table 1.

**Table 1:** Application of analog platforms in sensorimotor and upper limb research.

Platform	PF	SAC	Neutral buoyancy	BWS	HDT/HUT	DI	HDBR	Axial loading suits
Duration scalability	20–30 s	Minutes–hours	Hours/session	Weeks–months	Hours–days	Days–weeks	Weeks–months	Continuous use
HG fidelity	~0 ≤ g ≤~ 1.8	GIA, >1 g	~0 ≤ g ≤ ~0.38 (mechanical unloading)	~0.2 ≤ g ≤~ 0.8 (low vestibular realism)	1 g	~0 g (support unloading)	~0 g (axial unloading)	≥1 g equivalent loading
Cardiovascular and musculoskeletal deconditioning	Low	Moderate	Low	Moderate	Moderate	Strong	Strong	Reduces deconditioning
Sensorimotor reweighting	Acute only	Partial	Limited	Task-specific	Acute	Rapid adaptation	Chronic reweighting	Abscent
Vestibular vs somatosensory mismatch	Quick transitions, conflicting cues	Linear acceleration ≠ tilt	Hydrodynamic ≠ g forces	Intact vestibular input	Vestibular vs. body axis mismatch	Altered support cues, preserved vestibular gravity	Vestibular invariance	Sensory invariance
Scalability	Low	Moderate	Moderate	High	Moderate	Moderate	High	High
Limitations	Costly, low repetition, small sample sizes, short duration	Complex logistics, limited availability, Coriolis effect, motion sickness	Require training	Limited application, complex	Short duration	Constraind movement	Costly, limited tasks	Device constraints, altered biomechanics

PF, parabolic flight; SAC, short arm centrifuge; BWS, body weight support; HDT/HUT, head-down tilt/ head-up tilt; DI–dry immersion; HDBR, head-down bed rest; GIA, gravitational inertial acceleration, ~approximately, ≠ not equal to, ≤ – less than or equal to ≥ − greater than or equal to, g – acceleration due to gravity (*g* = 9,81 m/s^2^). Color coding: Green = hight suitability, Yellow = moderate suitability, Red= Low suitability.

*Gravity magnitude modulators* alter gravitational acceleration and impact otolith input and body–limb torques, e.g., PF, body-weight support (BWS), centrifuges, and gravity compensation systems. PF involve studies of upper-limb tasks, with limited experiment duration and high cost. Current BWS, initially developed for post-stroke rehabilitation, provides long-duration assessments for space training (De Martino et al., 2023). For example, the Variable Gravity Suspension System reduces effective body weight during walking, running, and jumping for astronauts (Swain et al., 2025). Other platforms, such as NASA’s ARGOS and ESA/DLR’s LUNA facility (German Aerospace Center (DLR), 2026), are well-suited for walking and running; however, there are currently no solutions for upper-body training under HG. Centrifuges, a common method in space medicine, have already demonstrated efficacy in clinical studies for stroke rehabilitation, improving gait, balance, and cardiovascular function (Kourtidou-Papadeli et al., 2023), and it can only be indirectly involved in upper-limb research. Gravity compensation systems, e.g., exoskeletons, are widely used for rehabilitation in stroke, spinal cord injury, traumatic brain injury, and upper-limb studies (Gull et al., 2020). While providing mechanical unloading for the upper limb, they do not alter vestibular input, as this limits perceptual reweighting.

*Terrestrial analogs of gravitational unloading* include head-down tilt (HDT), which simulates the vestibular fluid shift experienced in MG toward the head and chest; head-up tilt (HUT) which mimic orthostatic stress after spaceflight effects by shifting fluid toward the legs through changes in orientation relative to gravity (Hoenemann et al., 2023); DI which provides an axial unloading model and support removal to simulate MG (Kozlovskaya et al., 2006); and HDBR which simulate vestibular fluid shifts, cardiovascular, musculoskeletal, and sensorimotor MG effects (Hargens and Vico, 1985). These models have been successfully applied in clinical contexts for early-stage rehabilitation, blood pressure treatment, cortisol treatment, and alleviating arthritic and trauma pain (Orlov et al., 2014). In addition, DI was used for symptomatic treatment in Parkinson’s disease (Meigal et al., 2022) and for decreasing muscle tone (Amirova et al., 2021). These analogs are limited in use for upper-limb studies due to inducing axial unloading or orientation changes rather than true alterations in gravitational torque and dynamic arm loading.

*Axial loading systems,* e.g., the « Pinguin » suit, rebalance the load on the body through elastic components aligned with anatomical sites and opposing muscle pairs, thereby optimizing joint movements, facilitating neuromuscular activation, and giving proprioceptive input (Kozlovskaya et al., 1995; Motanova et al., 2022). These suits have now been directly used in rehabilitation. Its adapted version, «Adeli», is used in rehabilitation to enhance postural control, vertical stability, gait recovery, and neuromuscular activation, improving gross motor function and balance in children with diplegic cerebral palsy (Semenova, 1997; Nemkova et al., 2000b; Nemkova et al., 2000a; Motanova et al., 2022). The «Regent» suit stimulates recovery after motor disorders caused by stroke and traumatic brain injury by improving gait and posture, increasing the intensity of proprioceptive input, and supporting neurorehabilitation (Motanova et al., 2022). Another example is the Variable Vector Countermeasure Suit, which integrates inertial units and miniaturized control-moment gyroscopes on body segments to generate viscous resistance during movements against a “down” direction, facilitating gait stabilization for astronauts and rehabilitation applications (Duda et al., 2015). Such systems do not change the gravitational load on the upper limb, making them unsuitable for studying performance and verticality perception.

Therefore, solutions for neurorehabilitation and human spaceflight converge on promoting adaptive sensorimotor recalibration under altered mechanical, sensory, and gravitational conditions, but they also have limitations and constraints. Further technological development is needed with a focus on adaptation processes and mechanisms, especially for studies on manual and/or upper-limb performance.

## 5 Discussion

There is evidence that astronauts experience significant sensorimotor changes in perceptual and motor processes, as well as adaptation during spaceflight missions, especially during and after g transitions, when the most critical operational tasks are conducted (Hilbig et al., 2017; Saveko et al., 2023; Kuldavletova, 2020; Alberts, 2016; Mergner, 2002; Bringoux et al., 2012; Ritzmann et al., 2015). Currently, NASA emphasizes manual spacecraft control, human adaptability, and its prediction, neural remodeling, integration of sensorimotor systems, and specialized training as key research interests (Bloomberg et al., 2015). Moreover, the ESA’s Explore 2040 strategy focuses on developing technological and scientific capabilities to provide safe and effective human performance during lunar and Martian surface missions (European Space Agency, 2026).

Significant gaps remain that impede the design of reliable, user-specific solutions for HG simulation and countermeasures. First, human data from prolonged exposure to HG (lunar/Martian) remain inaccessible but are critical for mission preparation, making it difficult to estimate safety margins. Second, it is necessary to identify thresholds for HG level, duration, and exposure frequency for training to ensure high task performance. Third, it is critical to develop solutions and validated countermeasures to achieve program priorities for future missions (De Martino et al., 2023; Shelhamer, 2021). To address this problem, these gaps must be filled, leveraging new potential directions for future research, specifically in upper-body investigation as a new research class, which is currently underexplored.

First, it is crucial to aim to design long-term studies in simulated altered gravity and practical conditions, defined tasks for missions. Findings from such studies can be directly applied to operational guidelines or used to develop technical solutions for long-term space missions. Also important to aim for gravity-variable conditions for upper-limb and fine motor control assessment, ideally using tool-based tasks.

Moreover, such studies should support adaptive systems with parallel motor and perceptual processes. It will require developing emergent solutions that integrate current sensorimotor knowledge into adaptive control systems capable of real-time assistance or facilitating training. Such solutions should be redesigned around human constraints, including postural caution, and long-loop sensorimotor control (Hilbig et al., 2017; Maillard, 2023a). They should be user-specific adaptive gravity systems based on real-time performance metrics and perceptual state.

Currently, task-relevant Human-Machine Interaction (HMI) can simulate real mission constraints. A promising solution is a configurable HMI with assistive load redistribution, which enables us to work with target-specific limbs, isolate loading components to test multiple hypotheses, and operate in real-time assistance. This provides more precise adaptive weight support and analysis of task performance. However, such an approach would require a large dataset for predictions. The dataset can be collected using one or a combination of the methods listed in Table 1. The following step is to combine these systems with predictive, person-specific models, complemented by AI/ML that adapt to each user’s cues and weighting, and assist with task-specific context in real time if an error or a shift is identified.

This solution can be validated by testing whether it increases tool or object manipulation performance and improves reaching compared with unsupported conditions. It can be quantified using accuracy, variability, movement time, and progress across sessions. The main benefit would be shown in long-duration, repeated-measures designs spanning days to weeks.

Under such projects, neuroscientists, engineers, biomechanics specialists, medical doctors, mission planners, and users should collaborate closely. In our experience, cross-disciplinary investigations still require some facilitation systems for such projects. Convergent space medicine and rehabilitation can generate translational data across these domains by leveraging similar tasks, the same measures, standardized outcome performance-related metrics, and measurement techniques, enabling labs to compare results, accelerate learning, and develop data-driven, real-time, synchronized, and personalized countermeasures.

Future upper-body investigations should move beyond isolated biomechanical unloading toward integrated, convergent systems that combine HMI technology with data-driven adaptive control. By embedding upper-limb studies within multimodal frameworks, research can more accurately characterize human motor and perceptual processes under variable gravitational loading, for both astronaut preparation and neurorehabilitation.

## Data Availability

The original contributions presented in the study are included in the article/supplementary material, further inquiries can be directed to the corresponding author/s.

## Footnotes

1 Spatial orientation — the brain’s sense of body position in external environment. Visual vertical perception — visual judgement of direction of true vertical. Proprioception and vestibular function — the body’s ability to sense position and movement through muscles, joints, and head motion, maintaining balance relative to gravity from the inner ear. Body schema — based on an internal brain representation of the body movement, position, size and shape.
